# Novel Rare Earth (RE)-Free Nanocomposite Magnets Derived from L1_0_-Phase Systems

**DOI:** 10.3390/nano13050912

**Published:** 2023-03-01

**Authors:** Alina Daniela Crisan, Ovidiu Crisan

**Affiliations:** National Institute for Materials Physics, P.O. Box MG-7, 077125 Magurele, Romania

**Keywords:** L1_0_ phase, nanocomposite magnets, crystallization, Mössbauer spectroscopy, structural phase transformation

## Abstract

In the quest for novel rare earth (RE)-free magnetic materials, which also exhibit other additional properties such as good corrosion resistance and potential to operate at higher temperatures, an alloy deriving from the binary FePt system, with Mo and B addition, has been synthesized for the first time, using the out-of-equilibrium method of rapid solidification form the melt. The alloy with the composition Fe_49_Pt_26_Mo_2_B_23_ has been subjected to thermal analysis through differential scanning calorimetry in order to detect the structural disorder – order phase transformation as well as to study the crystallization processes. For the stabilization of the formed hard magnetic phase, the sample has been annealed at 600 °C and further structurally and magnetically characterized by means of X-ray diffraction, transmission electron microscopy, ^57^Fe Mössbauer spectrometry as well as magnetometry experiments. It has been proven that after annealing at 600 °C the tetragonal hard magnetic L1_0_ phase emerges via crystallization from a disordered cubic precursor and becomes the predominant phase in terms of relative abundance. Moreover, it has been revealed by quantitative analysis via Mössbauer spectroscopy that the annealed sample exhibits a complex phase structure, where the L1_0_ hard magnetic phase is accompanied by few other soft magnetic phases, in minority abundance: the cubic A1, orthorhombic Fe_2_B and residual intergranular region. The magnetic parameters have been derived from 300 K hysteresis loops. It was shown that, contrary to the as-cast sample which behaves as a typical soft magnet, the annealed sample presents strong coercivity and high remanent magnetization, accompanied by a large saturation magnetization. These findings offers good insight into the potential developing of novel class of RE-free permanent magnets, based on Fe-Pt-Mo-B, where the magnetic performance emerges from the co-existence of hard and soft magnetic phases in controlled and tunable proportions, capable of finding good applicability in fields requiring good catalytic properties and strong corrosion resistance.

## 1. Introduction

There is nowadays a strong interest for multipurpose materials, specifically designed for various applications. For instance, researching for novel RE-free nanocomposite magnets has yield the discovery of several classes of magnets that are derived from systems bearing formation of L1_0_ phases. The tetragonal L1_0_ phase is the high ordered crystal structure which in the case of FePt-based alloys gives its hard magnetic features. Fe-Pt binary system and their derived FePt-based alloys usually crystallizes in a disordered face-centered-cubic fcc A1 structure. The fcc structure transforms after appropriate annealing into an ordered face-centered-tetragonal fct allotrope phase which is of interest since it exhibits high coercivity and large magnetocrystalline anisotropy [1]. L1_0_-phase magnets present equally good magnetic performance in terms of energy product, being also possible to perform at higher temperatures than classical RE magnets, and have good corrosion resistance properties. The nanocomposite magnets derived from FePt system is widely considered as potential candidates for applications requiring high temperatures of operation [1,2,3,4,5,6] with potentially good resistance to corrosive media. The binary FePt nanoparticles have also strong catalytic response, being also a material with significant biocompatibility. There have been published studies regarding their applicability in biomedical fields [1,2,3], as catalytic materials [4], sensing and biosensing [5,6]. For nanoparticles preparation some varied techniques have been used ranging thermal decomposing of precursors beyond their boiling temperatures [7], reduction of Pt acetylacetonate [8], reverse micelles [9], microwave irradiation [10], sonochemical ways [11], plasma methods [12], or gas phase techniques [13]. This allows producing FePt in its nanoparticulate form, with high yield rate, finely formed shapes, and diameters dictated and tuned by synthesis conditions. With respect to catalytic properties, some other ternary systems such as Fe-Mo-B have shown good perspectives [14,15,16] therefore a mixed stoichiometry compound deriving from FePt systems with Mo and B addition can presumably also yield good magnetic and catalytic properties. There have been also some other works which provided a good insight into the influence of preparation conditions and the specific magnetic parameters obtained, in a wider range of magnetic alloys and films [17,18,19,20,21,22,23,24,25,26]. Top applications are targeted by the use of the FePt-derived systems. FePt auto-arranged on templates such as the block copolymers [27] are suitable for applications in the field of magnetic recording media [28,29]. If the FePt and FePt-derived systems are considered in their bulk form, either powders, melt spun ribbons or cast alloys, they have good hard magnetic properties: high T_C_, large uniaxial anisotropy, very resistant to corrosive media, and high enough energy product. From elastic and mechanical point of view, the FePt-derived compounds may exhibit good hardness properties, as shown by some nanoindentation studies [30]. The pathways for synthesis that are out-of-equilibrium thermodynamically, such as the rapid solidification form the melt [31] or the mechanical alloying method [32] are showing to be flexible enough as they allow good yield in terms of quantity as well as neatly enough control of the stoichiometry, especially if ternary or quaternary alloys based on the initial FePt system are needed.

Motivated by the possibility to obtain a new class of RE-free magnet, derived from the FePt system, and at the same time by the possibility to obtain good catalytic properties, we had the idea to synthesize a quaternary Fe-Pt-Mo-B alloy, by taking advantage on both our previous expertise in developing FePt-based magnets [24,25], as well as on previous results obtained in Fe-Mo-B systems [14,15,16]. In the present work we report on the synthesis for the first time of a quaternary alloy Fe-Pt-Mo-B, by using a rapid solidification from the melt method. We also report on their structural and magnetic features and we show the formation of hard magnetic L1_0_ phase in our alloy, upon appropriate annealing of the as-obtained melt spun ribbons.

## 2. Materials and Methods

### 2.1. Synthesis

The metallurgical way of preparation of our quaternary alloy, the rapid solidification from the melt method, was considered for two main reasons. One of the reasons is linked to the needed formation of the desired L1_0_ phase. While FePt-based alloys normally forms the face centred cubic A1, disordered phase, the melt spinning method can translate into the solid state, a phase structure of an alloy which can be only present in the liquid state. By this method, metastable phases such as the L1_0_ phase, manifesting in the molten state, can be obtained directly by fast solidification of the melt, in the solid state as well. It is to be mentioned that in a similar quaternary FePt-based alloy, direct formation of the L1_0_ phase was obtained, from the as-cast state, without the need of post-synthesis annealing [31]. To maximize the amount of L1_0_ phase formation, based on previous results [24,25], we have seen than adding a few percents of refractory metal is desirable, as it promotes early ordering in the cubic phase and occurrence of tetragonal L1_0_ phase is secured. The other reason is linked to the high yield of the preparation method. Using the rapid solidification from the melt, long, continuous and homogeneous metallic ribbons, with constant thickness are obtained. This is particularly handy if it comes to envisaging their use in industrial applications such as for instance in transformer cores or for oxygen evolution reactions, if the alloy is to be used for its catalytic properties. 

Taking all these into account, a quaternary alloy with composition Fe_49_Pt_26_Mo_2_B_23_ has been synthesized by the rapid quenching method, where elemental metals are mixed and melted then purged on a high velocity rotating copper wheel to fast quench and form the metallic ribbons. The mixed alloy, with the above-mentioned stoichiometry, was made with flakes from Alfa Aesar GmbH, Karlshruhe, Germany, with 99.9+% purity. These mixed flakes were inserted into a quartz crucible, which is situated in a high vacuum deposition chamber (Melt Spinner MSP60 from Edmund Bühler GmbH, Bodelshausen, Germany) then heated up to 1300 °C in order to melt the flakes. The process is 3 times repeated in order to homogenize the whole alloy. After the first melting, the alloy was slowly quenched and then the temperature was raised again to 1300 °C and slowly quenched again. During this time, a melt stirrer has been used to ensure alloy homogenization. Then, obtained melt is flown via a 5 mm bottom nozzle onto the rotating copper wheel (45 m/s). The fast rotation ensures ultra rapid quenching of the purged molten alloy at a rate of about 10^6^ K/s. For the purpose of forming the phases of interest, the obtained as-cast ribbons have been annealed in vacuum controlled induction oven. For annealing, the heating conditions were chosen to be 600 °C (873 K) for 1 hour with a heating rate of 5 K/min followed by the cooling down to room temperature, all in vacuum conditions (10^−5^ mbar). 

### 2.2. Characterization

The samples prepared as described above have been structurally and magnetically characterized using a plethora of characterization techniques. The true stoichiometry of the as-cast samples has been verified using Energy-Dispersive X-ray Spectroscopy (EDS-EDX). The temperature evolution of the sample structure was checked using the differential scanning calorimetry (DSC) whereas the structure and morphology of the as-cast and annealed samples were analyzed using X-ray diffraction (XRD), transmission electron microscopy (TEM) and Mössbauer spectroscopy (MS). 

The samples morphology and microstructure were investigated using a high-resolution electron microscope JEOL JEM ARM 200F (from JEOL-Europe, BV, Zaventem, Belgium) operated at 200kV. The thinning of the specimens prepared for the microscopy studies were prepared in a geometry with plan-view orientation, using the ion milling facility at a 7° angle of ion beam incidence and at a 4 kV accelerating voltage, by using a Gatan PIPS facility (from Ametek SAS, Elancourt, France). The electron microscope is dotted with an EDX module that was used for checking the true composition of the sample.

DSC thermal analysis has been performed for the fast quenched, as-cast alloy by uniformly heating the samples in the DSC crucible analyser between 300–650 °C (573–923 K) with a heating step rate of 10 K/min. DSC thermo-analysis has been performed with a Setaram DSC 111 system from KEP Technologies, EMEA, Caluire, France, in the differential mode, under continuous Ar flow.

The structural features of both the as-cast and the annealed melt spun ribbons were evidenced by XRD analysis using an instrument D8 Advance from Bruker AXS GmbH, Karlsruhe, Germany. All the samples diffractograms were acquired in a θ–2θ geometry using the Cu Kα wavelength radiation λ = 1.54 Å and afterwards were subsequently analyzed with full-profile Rietveld-type analysis, powered by MAUD (Material Analysis Using Diffraction) software, from University of Trento, Italy [33]. Besides straightforward identification of the nature of the crystalline phase by Bragg peak identification, other parameters derived from the XRD data, through the use of this full-profile analysis, such as the lattice parameters and the average grain sizes were obtained. Besides XRD, another technique which allows quantitative analysis of the nature and the abundance of the Fe-containing phases, albeit amorphous or crystalline, as well as their electronic properties, is the Mössbauer spectroscopy. The samples, as-cast and annealed, were analyzed using ^57^Fe Mössbauer spectroscopy performed at 300 K with a linear Mössbauer spectrometer dotted with a bath cryostat, from Oxford Instruments, Abingdon, UK, at 300 K, in a conventional transmission geometry set-up. The gamma ray source for the Mössbauer analysis was a ^57^Co source in Rh matrix. Magnetometry studies were done for a complete image of the magnetic state and identification of optimal magnetic parameters. The magnetic features were obtained with a Superconducting Quantum Interference Device (SQUID). This device enables acquiring the hysteresis loops in an applied field of up to 5 T, either parallel or perpendicular to the sample plane, at various temperatures ranging from 1.5 to 300 K. 

## 3. Results and Discussion

### 3.1. Composition and Morphology

The energy-dispersive X-ray spectroscopy (EDX) module of the transmission electron microscope JEOL was employed for determining the real stoichiometry of the prepared as-cast sample. The chemical composition analysis is based on the identification of characteristic X-ray emission lines tabulated for EDX analysis. The emission lines of Fe and Pt have been well identified and therefore the relative concentration of these elements has been accurately calculated. In addition, even though the M line of Pt (E = 2.048 keV) the Lα line of Mo (E = 2.293 keV) are spectroscopically close, we have been able to correctly deconvolute these lines, taking also into account the low concentration of Mo, and therefore we could calculate correctly the amount of Mo. Due to the difficulties of obtaining adequate quantification of light elements such as boron, this being a known limitation of the EDX spectroscopic technique, we considered the composition of boron to be the same as the nominal one. The measured sample stoichiometry is given in Table 1. It was found that the measured composition of the sample is quite close to the nominal one, within an error of 1.2 at%. The root mean square deviation, averaged over all three determinations, was about 0.18 at%.

### 3.2. Thermal Analysis

The thermal analysis using the differential scanning calorimetry has allowed us to determine the heat flow exchanged with the system during the constant annealing of the sample in a wide temperature range from room temperature up to 650 °C (923 K). This study is useful in the identification of any change in energy associated with a structural and/or magnetic phase change or phase transition. In particular, in our case, where FePt is expected to undergo a structural phase transformation from cubic A1 to tetragonal L1_0_ structure, such study could come in handy for the estimation of the onset value of temperature at which this structural phase transformation may occur. The sample exchanges even more energy with the exterior, during the DSC experiment, if it undergoes an amorphous-to-crystalline structural change. This crystallization effect is more visibly recorded due to the large exothermic effect associated to the atomic ordering during the sample crystallization from an initially amorphous precursor. 

**Figure 1:**
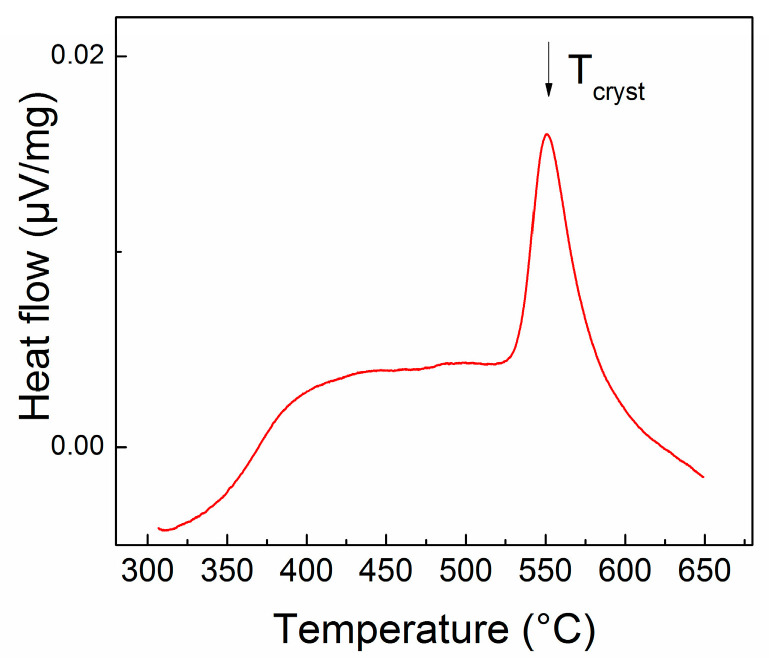
DSC curve plotted between 300 °C and 650 °C for the as-cast sample.

Figure 1 shows the DSC curve, where the heat exchanged by the sample is plotted versus the heating temperature, recorded during linear heating from 300 °C up to 650 °C, with a heating rate of 10 K/min. 

It can be seen that there is a slow increase in the heat flow between 300 °C until around 400 °C before the flow reaches a plateau lasting until about 530 °C. Beyond this temperature the system records a quite sharp exothermic peak with a maximum centered at about 550 °C, exothermic peak that shows a massive heat exchange with the exterior. At about 650 °C the system reaches the reference flow value of zero, signifying that the exothermic process associated with that peak has ended. In agreement with previous reported data on FePtB-based alloys [24,25] where it has been observed that the initial as-cast state is an amorphous-like solid solution, until 450 °C–500 °C, we have interpreted this peak as originating from the massive heat exchange associated with the sample crystallization from its initial structurally disordered state. Moreover, as the temperature of crystallization is quite close to the value of the disorder—order structural phase transformation with formation of the L1_0_ tetragonal phase, it is conceivable that the large exothermic effect due to the crystallization encompass also the much smaller exothermic effect associated to the structural A1 to L1_0_ phase transformation, occurring at almost similar temperatures, in this case. These assumptions will gain further confirmation from the XRD data, analyzed below.

### 3.3. Structural Analysis

We have furthermore used the results from the DSC measurement and we have chosen as annealing temperature the temperature that is beyond the crystallization peak, recorded at 550 °C. For this reason we have chosen to anneal the sample at 600 °C for 1 hour, in order to observe the structural changes induced by the phase transformation observed in the DSC. The annealing lasted for 1 hour in order to stabilize the new crystal structure formed beyond 550 °C and to develop larger areas or grains of tetragonal L1_0_ phase. 

**Figure 2:**
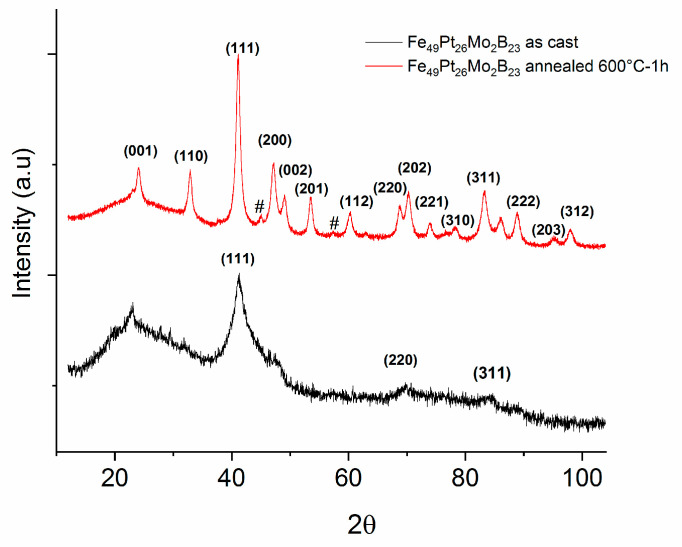
X-ray diffractograms of as-cast and annealed Fe-Pt-Mo-B sample.

Figure 2 shows the X-ray diffractograms recorded for both the as-cast and for the sample annealed at 600 °C for 1 hour. The diffractogram of the as-cast sample presents features that are typical for amorphous-like and nanocrystalline materials. The diffractograms present Bragg lines which are extremely broad but sufficiently well formed. The Bragg line broadening, typically encountered in chemically disordered amorphous-like phases, is observed together with the features of nanocrystalline A1 cubic symmetry within the sample. This implies that the as-cast state consists of a mixture of chemically disordered atomic arrangements, together with small nanocrystals with a certain degree of A1 cubic symmetry formed inside the amorphous matrix. The indexation of the observed Bragg reflections allows the identification of the crystalline phase as well as the nanocrystals mean size. The Bragg lines, marked on the graph, were unambiguously associated to the (111), (200), (220) and (311) *hkl* planes of the cubic symmetry. The peak analysis, using a Rietveld-type refinement technique has allowed us to prove that the observed Bragg peaks belong to the FePt face-centered-cubic (fcc) lattice (A1 phase, *Fm3m* space group, lattice parameter: a = 0.38 nm), an average size of 4 nm, a symmetry that is commonly observed in the case of colloidal near-equiatomic FePt nanoparticles [7,9]. 

In the diffractogram obtained for the sample annealed at 600 °C for 1 hour, there are notable differences from the as-cast sample. Several additional Bragg lines are observed, much narrower, witnessing for the fact that the sample is in a fully crystallized state, thus confirming our assumptions regarding the origin of the exothermic effect observed at 550 °C. Most of the additional Bragg peaks were indexed as belonging to the face-centered-tetragonal (fct) L1_0_ FePt phase (*P4*/*mmm* space group), with the exception of two peaks of very low intensity, marked on the graph with #, which are attributed to the orthorhombic Fe_2_B phase. The L1_0_ phase lattice parameters, calculated from the full-profile analysis were found to be: a = 0.385 nm and c = 0.317 nm, with an ordering parameter c/a = 0.823) and the average L1_0_ grain size being calculated to be about 18 nm. There is some overlapping of the main Bragg peaks of A1 and L1_0_ FePt phases, for instance fcc (111) and fct (111), however upon formation of the L1_0_ phase, the fcc (200) undergoes a tetragonal splitting with the formation of (200) and (002) Bragg peaks of the fct phase. More important, we have noticed the two Bragg peaks occurring at around 24° and 33° in 2θ, which constitute the well-known signature of the tetragonal phase, i.e. the so-called “superlattice” Bragg peaks (001) and (110). These peaks were unambiguously observed and indexed in the spectrum and indicate without doubt the occurrence of the tetragonal L1_0_ FePt phase in the annealed sample. The occurrence of two other Bragg lines, of lower intensity, attributed to the Fe_2_B phase, indicates the multi-phase character of the annealed sample. The formation of the Fe_2_B, co-existing with the L1_0_ FePt phase in the annealed sample, will be furthermore confirmed by Mossbauer spectroscopy studies.

**Figure 3:**
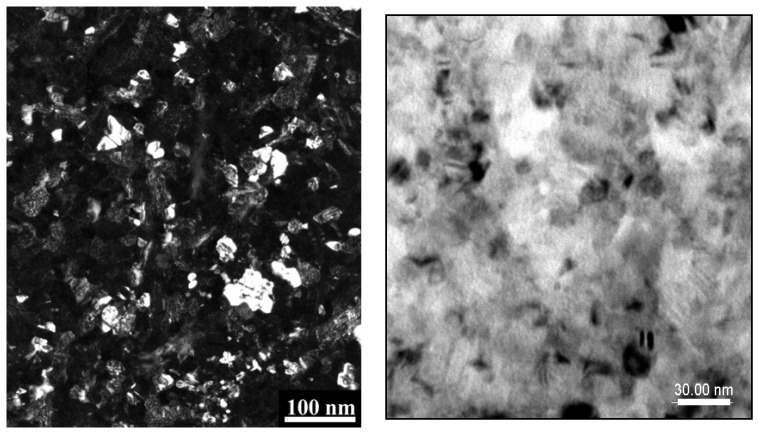
Transmission electron micrographs of two typical, granular area of the Fe-Pt-Mo-B annealed sample. Left hand image is the dark field mode TEM while the right hand image is the bright field mode TEM.

The granular structure of the annealed sample has been furthermore investigated using direct imaging by transmission electron microscopy. The TEM images have been taken with a 200 kV acceleration voltage and 2 nm beam resolution. Some typical examples of the microstructure observed in TEM are shown in Figure 3. The samples to image were thinned down to be transparent to the electron beam, by reactive ion etching by plasma discharge, following the procedure described in Section 2.2.

A microstructure consisting of well-defined, almost regularly dispersed, faceted grains with different image contrast is observed in this sample. The contrast difference indicates the multiple phase character of the observed grains and it is likely that the grains with strained top surface and those which give brighter contrast would be the FePt tetragonal grains, since Pt is the heavier element and the contrast is proportional to the Z number of the elements. The diffuse, out of focus, dark regions may belong to the residual boron-rich phase, as identified in XRD, boron being a much lighter element which does not furnish strong contrast under the electron beam. 

Among the well-dispersed grains, some large clusters of grains are also observed. These are not large bulk-like forms, but more probably, clusters of grains that coalesce one near the other on the linear facets of individual grains. By direct measurement of only the grains with visible edges, the mean grain size averaged over the visible grains in the images was determined to be about 16 nm. Consider the fact that only a small part of the microstructure, visible on the sample surface after etching, is imaged by TEM, therefore the results obtained by the hystographic method are highly local. As opposed to that, the grain size obtained from the full-profile analysis of XRD, which is a global analysis and the value for the mean grain size is averaged over the whole sample volume. Viewing these arguments, one many note that the agreement of the two results coming from these different investigations (16 nm by TEM and 18 nm from XRD) is truly remarkable. 

### 3.4. Mössbauer Analysis

**Figure 4:**
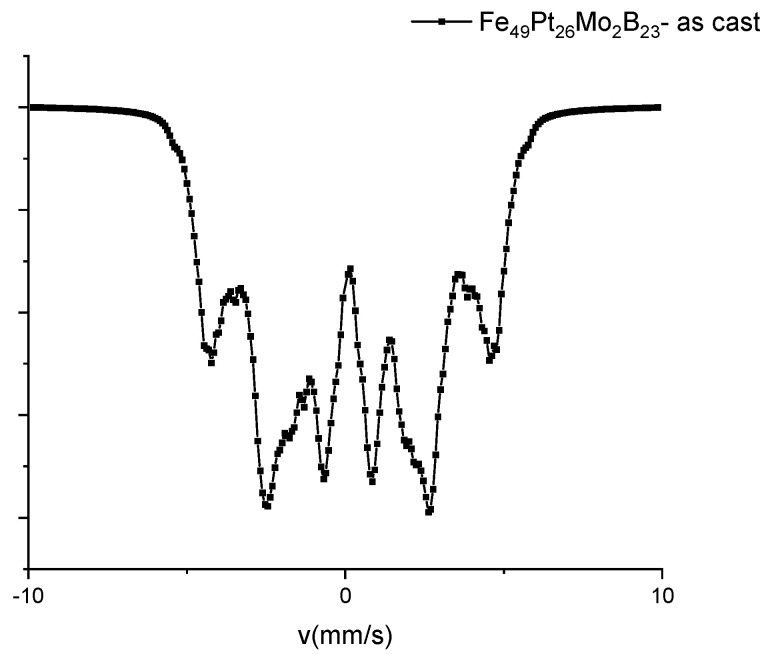
300 K Mössbauer spectrum of the as-cast sample.

**Figure 5:**
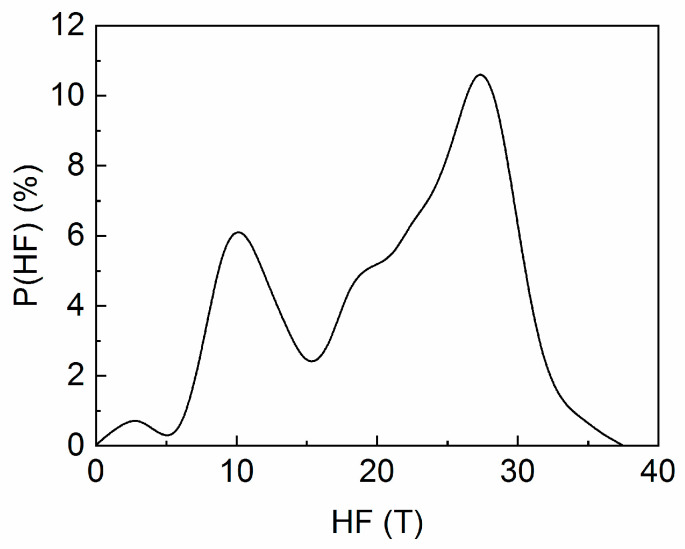
Bimodal hyperfine field distribution, as resulted from fitting of the Mössbauer spectrum in Figure 4.

In order to confirm the presence of all the identified phases in the annealed sample and to obtain valuable information regarding their abundance and electronic properties, a ^57^Fe Mössbauer spectroscopy investigation has been undertaken on both as-cast and annealed samples. Figure 4 depicts the 300 K Mössbauer spectrum of as-cast sample, which presents a broad, distributed sextet which is typical for amorphous-like Fe chemical environments. Such features of the Mössbauer spectrum has been also found in a large number of magnetic amorphous Fe-based alloys [18,19,25,26]. This shape of the spectrum confirms well the XRD results where structural disorder has also been documented in the as-cast sample. We have applied a model of fitting with a discrete distribution of magnetic sextet, with hyperfine fields (HF) ranging from 0 to 40 Tesla, accounting for the distributed Fe environments, as is the case in amorphous Fe-based alloys. In the Figure 4, the experimental data are shown by dots and the resulting fit using the above-described HF distribution is shown by the continuous line. The distribution of the hyperfine fields, as resulting from the fit is shown in Figure 5. It is seen that the spectrum fits very well with a bimodal HF distribution. The low HF mode, of smaller abundance, centers at around 11 T and the high HF mode, of higher abundance, centers at around 28 T. The bimodal character suggests that there are two predominant components of Fe environment. The high field component is assigned to Fe sites in environments that are rich in Fe, as is the case, for instance, of the disordered FePt phase. The low field component is assigned to Fe sites in environments that are poor in Fe, such as the case of intergranular regions, which are rich in B and Mo. From the values obtained for the HF of each mode (11 T and 28 T) we can assign the low field mode to a disordered, intergranular precursor, from which, after annealing, the orthorhombic Fe_2_B can be formed, while the high field mode can be attributed to the disordered cubic FePt, from which, after annealing, the ordered tetragonal L1_0_ will be formed. 

**Figure 6:**
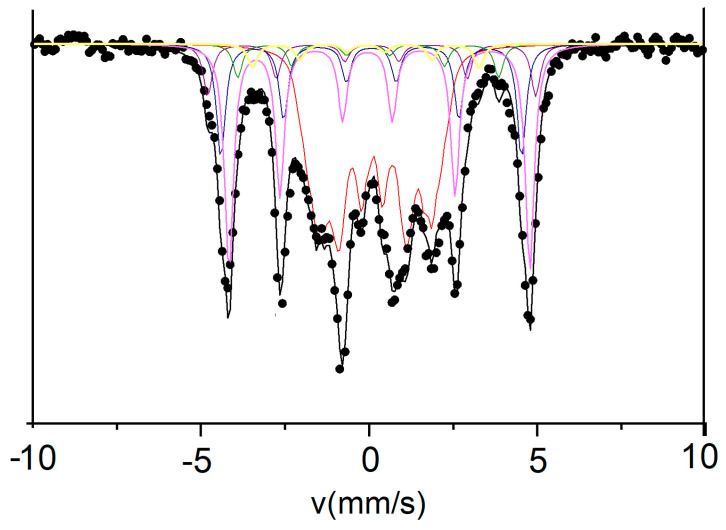
300 K Mössbauer spectrum of the annealed Fe-Pt-Mo-B sample.

The Mössbauer spectrum of the sample annealed at 600 °C for 1 hour (Figure 6) shows quite different features. The spectrum presents itself as a magnetic distributed sextet, but with much narrower lines. Here, lines 1 and 6 of the sextet are more intense and much better defined than in the case of the as-cast sample. The asymmetrical line profiles suggest that the sample is multi-phased, since there are contributions from various sublattices, convoluted, visible in the spectrum. The large, irregular shape of the transmission pattern, recorded between −2 mm/s and +3 mm/s suggests a contribution with low hyperfine field, probably due to the existence of a disordered residual matrix, with low Fe content, situated perhaps in the intergranular regions. 

Considering that heavy noble metals such as Pt exhibit a quite large X-ray absorption, also taking into account the Pt abundance in the sample stoichiometry, it was surprising to reach such a clearly resolved spectrum with strong enough line intensities. We have successfully fitted the Mössbauer spectrum with 6 different magnetic sublattices, in good agreement with the microstructural findings. The results of the fitting are listed in Table 2.

Contrary to the model of fitting from the as-cast state, where we have employed a model with continuous HF distribution, ranging between 0 and 40 T, here, due to the strong crystallinity of the samples and due to the overall shape of the experimental spectrum, we have chosen a finite number of magnetic sublattices with all hyperfine parameters considered free (not fixed) during the fitting, with the exception of the linewidth Γ/2, fitted but kept the same for the sublattices belonging to the same magnetic phase. The fitting provided very good results with the model we assumed. We have found a high field sublattice with a hyperfine field of 30 T and a quadrupole splitting value close to 0. Due to the high value of HF combined with the zero value for the QS, suggesting a cubic symmetry of this Fe chemical environment, we deduced that this component is attributable to the cubic FePt fraction, remained untransformed into tetragonal phase, at this stage of annealing. This cubic fraction amounts to 7%. For the following two contributions, with slightly lower hyperfine fields, the fitting provided an isomer shift (IS) value of 0.27–0.28 mm/s (relative to metallic Fe), quadrupole splitting (QS) value of 0.34–0.36 mm/s and hyperfine fields (HF) of 27.6–27.8 T. Judging by the large values of quadrupole splitting suggesting either tetragonal or a tetragonally-distorted cubic symmetry, coupled to the HF values smaller than in the case of the cubic A1 phase, we have attributed these two components to the tetragonal L1_0_ FePt phase, in agreement with findings from other works [34]. This tetragonal phase amounts to 52% abundance in the sample. The following two components (red and yellow) have smaller HF, and their values, 23.5 T and 20.9 T, are typical for the orthorhombic Fe2B phase, in full agreement with the findings from XRD data. The boride phase amounts to about 7%. The last component with the lowest HF of 9.8 is typical for disordered, paramagnetic-like chemical environment for the Fe and, from the obtained values of IS and QS we conclude that this component has a cubic symmetry and can be attributed to the residual, low in Fe, environment corresponding perhaps to an intergranular region enriched in non-magnetic elements (Pt, Mo, B). This indicates that the annealing we performed did not transform completely the cubic FePt phase into tetragonal L1_0_ phase, however we have managed to obtain a microstructure made of both hard (L1_0_) and soft (A1, Fe_2_B) magnetic phases which co-exist in the sample, thus giving rise to a potential exchange coupled, hard-soft magnet. This magnetic character is evidenced in the next section.

### 3.5. Magnetic Properties

**Figure 7:**
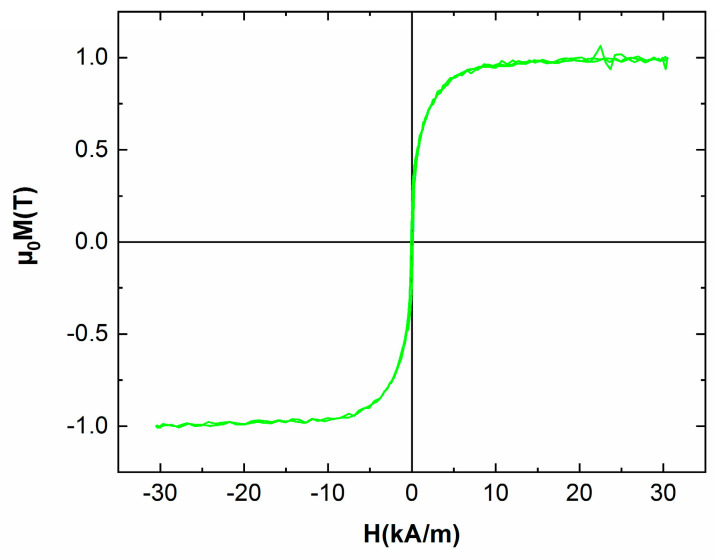
300 K hysteresis loop for the as-cast sample.

**Figure 8:**
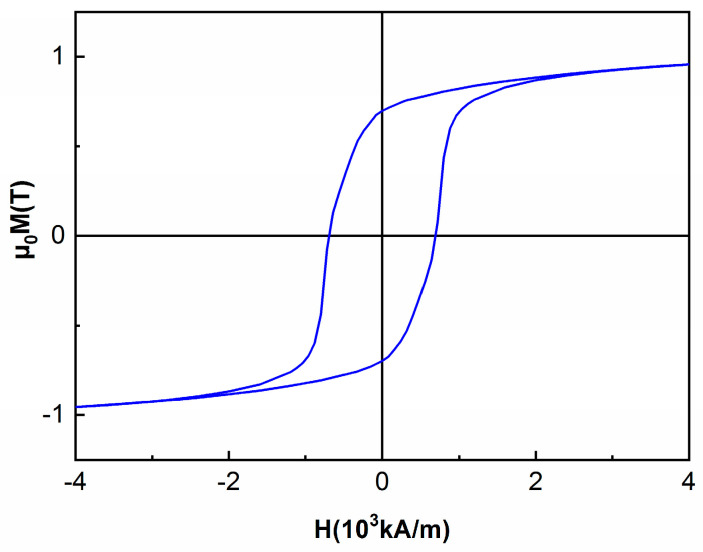
300 K hysteresis loop for the annealed sample.

The room temperature hysteresis loops have been recorded for both the as-cast and the annealed samples, in an applied field of up to 4 T, parallel to the ribbons plane. It can be seen that the hysteresis loop for the as-cast sample (Figure 7) shows a sharp rise to saturation of the magnetization, attaining its maximum at high values (1 T) that are typical for strong soft magnetic materials. The coercivity is almost zero which also accounts for features characteristic to a soft magnetic material. Correlated with the results from XRD, the magnetic response is consistent with the fcc A1 FePt phase which is known to be soft magnetic. On, the contrary, the sample annealed at 600 °C for 1 hour, shows a quite large hysteresis, of about 700 kA/m, and high value of saturation magnetization, of about 0.9 T (Figure 8). 

Moreover, the high coercivity combined with a large saturation magnetization is fully consistent also with the findings from the Mössbauer analysis where the co-existence of several hard and soft magnetic phases in various abundances has been confirmed. This creates good premises that a good exchange coupled nanocomposite magnet can be obtained also in the Fe-Pt-Mo-B system.

It is known that the figure of merit of any good magnet is given by its maximum energy product, (BH)_max_, equivalent to its capacity to store magnetic energy during any magnetization—demagnetization process involving its use. The maximum energy product can be easily calculated from the obtained hysteresis loops as follows. 

**Figure 9:**
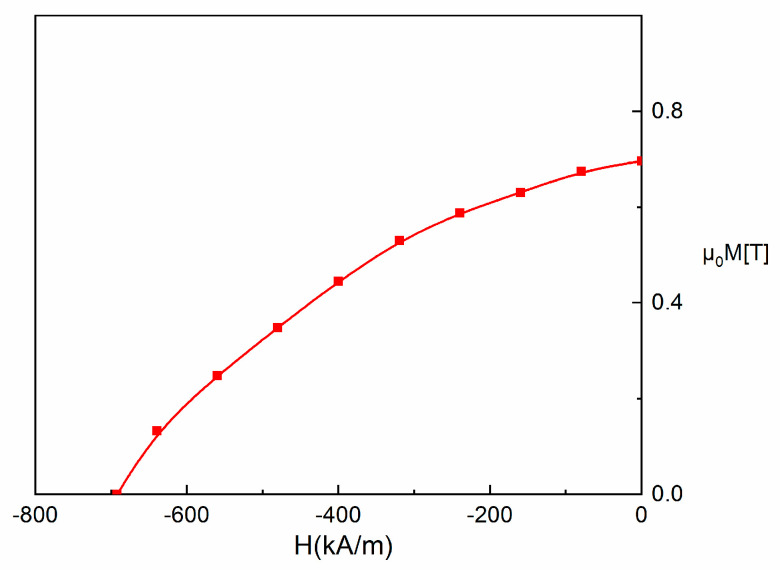
Demagnetization curve plotted from the hysteresis loop for the annealed sample.

**Figure 10:**
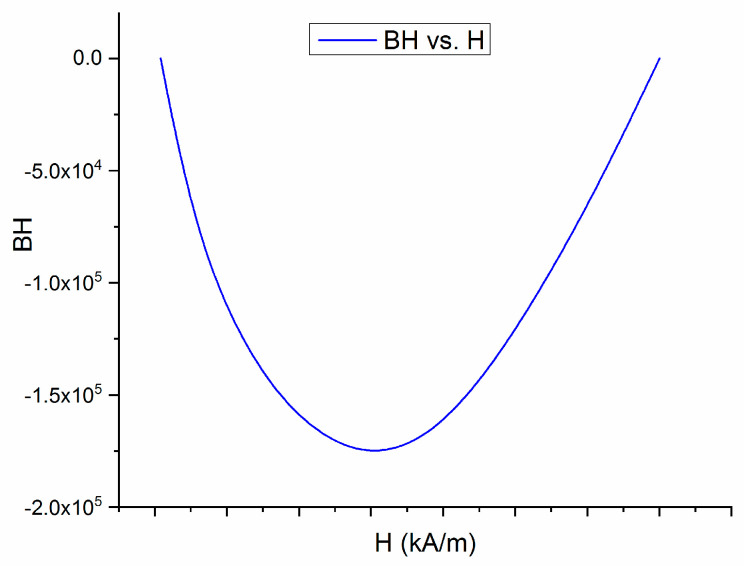
BH vs H plot as obtained from the demagnetization curve.

The fourth quadrant of the hysteresis loop for the annealed sample is plotted in Figure 9. It represents the demagnetization curve of the sample, i.e. the change of magnetization when the applied field is opposite in sign and the material diminishes its overall magnetization starting from the remanence point (around 0.7 T) until it reaches zero value, at the negatively applied field equal to the coercive field of the sample (around 700 kA/m). The experimental points in the fourth quadrant are then used to calculate BH and then plot this product versus the applied field. The BH vs. H plot is given in Figure 10. This plot shows a peak at a value calculated to be about 79 kJ/m^3^ (10 MGOe) and this is denoted as (BH)_max_, the maximum energy product. 

This good value of the energy product equals the values for other FePt and CoPt bulk alloys, reported in the literature [35]. These results show that there are good premises for further development of such Fe-Pt-Mo-B alloys in view of potential applications as RE-free magnets.

## 4. Conclusions

An alloy derived from FePt binary system, where Mo and B has been added with the purpose of obtaining an exchange coupled magnet with good magnetic performance and potentially good additional properties such as resistance to corrosion and catalytic properties. The idea is to combine the good hard magnetic properties of FePt, a material which is also studied for its catalytic potential, with the good soft magnetic properties of Fe-Mo-B, which is also a good catalytic material. The material has been prepared using the ultrarapid quenching from the melt and fully characterized, structurally and magnetically. While in its as-cast state, the sample shows chemically disordered cubic A1 symmetry, as revealed by XRD and Mössbauer studies, after annealing at 600 °C the sample undergoes a disorder-order phase transition during its crystallization from the as-cast disordered state. This transition has been documented by DSC thermograms and its results have been thoroughly documented by XRD and Mössbauer analysis. It was shown that the annealing gave rise to multiple hard and soft magnetic phases co-existing in the sample. While XRD showed the formation of tetragonal L1_0_ FePt hard magnetic phase together with some amounts of orthorhombic borides, the quantitative Mössbauer analysis allowed us to accurately map the phase structure. Concretely, it was proven that in the annealed sample, the tetragonal L1_0_ phase is the most abundant with 52% relative content and it co-exist with some untransformed fraction of cubic A1 FePt and orthorhombic Fe_2_B in lower amounts (7% each) and a residual intergranular phase, lower in Fe content, as proven by the low HF value, of about 34% abundance. All these compounds were unequivocally identified via the magnetic sublattices derived from fitting of the Mössbauer spectrum, each with typical isomer shift, quadrupole splitting and hyperfine field values. Hysteresis loops recorded at 300K confirmed the structural findings and showed strongly increased coercivity due to the granular mixing of hard magnetic L1_0_ phase with soft magnetic phases. Values that are comparable with best magnets from the FePt bulk alloy classes have been recorded: coercivity of about 700 kA/m, remanent magnetization of about 0.7 T and saturation magnetization of 0.9 T. From the fourth quadrant of the hysteresis loop, the BH product has been derived and it was calculated that the maximum energy product (BH)_max_ reaches about 79 kJ/m^3^ a value comparable with other FePt-based magnets. We have thus shown for the first time that the quaternary Fe-Pt-Mo-B melt spun alloy has strong perspectives to be used as a novel class of RE-free nanocomposite magnet, with potentially good corrosion and catalytic properties.

## Figures and Tables

**Table 1:** The measured sample composition, as revealed from EDX analysis of the as-cast sample.

Nominal Composition (at.%)	Fe (at.%)	Pt (at.%)	Mo (at.%)	Measured Composition (at.%)
Fe_49_Pt_26_Mo_2_B_23_	47.8	26.8	2.4	Fe_47.8_Pt_26.8_Mo_2.4_B_23_

**Table 2:** The fitting results of the Mössbauer spectrum of the annealed sample, assuming the fitting model with 6 discrete magnetic sublattices, color coded as in the Figure 6.

300 K					
IS (mm/s)	Γ/2 (mm/s)	QS (mm/s)	HF (T)	%	Magnetic Phase
**0.24**	**0.16**	**0**	**30.0**	**7**	**fcc FePt A_1_**
**0.27**	**0.16**	**0.34**	**27.6**	**19**	**fct FePt L1_0_**
**0.28**	**0.16**	**0.36**	**27.8**	**33**	**fct FePt L1_0_**
**0.13**	**0.16**	**0**	**23.5**	**4**	**Fe_2_B**
**0.06**	**0.16**	**0**	**20.7**	**3**	** Fe_2_B**
**0.26**	**0.17**	**0.03**	**9.8**	**34**	**Residual cubic**

Estimated errors are: 0.02 mm/s for IS, 0.02 mm/s for QS and 0.1 T for HF.
